# Minoritised ethnic women’s experiences of inequities and discrimination in maternity services in North-West England: a mixed-methods study

**DOI:** 10.1186/s12884-022-05279-6

**Published:** 2022-12-22

**Authors:** Gill Thomson, Julie Cook, Nicola Crossland, Marie-Clare Balaam, Anna Byrom, Raeesa Jassat, Sabina Gerrard

**Affiliations:** 1 School of Community Health & Midwifery, University of Central Lancashire, Preston, Lancashire PR1 2H2 UK; 2 Applied Health Research Hub, University of Central Lancashire, Preston, Lancashire PR1 2H2 UK; 3 School of Medicine, University of Central Lancashire, Preston, Lancashire PR1 2H2 UK; 4 School of Nursing, University of Central Lancashire, Preston, Lancashire PR1 2H2 UK

**Keywords:** Minority ethnic, Maternity care, Mental health, Mixed-methods, Discrimination

## Abstract

**Background:**

Minoritised ethnic perinatal women can experience judgemental and stigmatising care due to systemic racism. Discriminatory care contributes to increased risks of poor maternal and infant outcomes, including higher rates of mental ill-health. This study aimed to explore minoritised ethnic women’s experiences of maternity services, including maternity care and mental health support, within a North-West England locality. Here we use an equity lens to report the findings that describe if and how women’s personal, cultural, and spiritual needs were met, their experiences of discriminatory and prejudicial care, and to identify recommendations for service provision.

**Methods:**

A mixed-methods study was undertaken comprising an online survey, interviews, and community consultations. Questions explored access to and experiences of antenatal care and education; information, communication, and choice; experiences of (dis)respect and judgement; mental health needs and support; cultural/religious needs and support; and overall experiences of maternity care. Eligibility criteria were: women, 18+ years, from self-reported minoritised ethnic backgrounds, who had given birth in the previous 2 years and received maternity care in the locality. Surveys were available in seven languages and distributed via social media, mother-baby groups, and community locations. English-speaking survey participants were invited to take part in a follow-up interview. Community staff were approached to collect data on behalf of the study team. Quantitative data were analysed descriptively (n, %) and merged with qualitative data into descriptive themes.

**Results:**

Overall, 104 women provided data; most self-identified as Asian (65.0%) or Black (10.7%) and were aged between 30–34 (32.0%) or 25–29 years (23.3%). Four descriptive themes are reported: ‘accessing care’ details variations and barriers in accessing maternity care; ‘communication needs, and resources’ describes views on adaptions and resources for specific communication needs; ‘meeting religious and cultural needs’ outlines how various religious and cultural needs were met by maternity providers; ‘discriminatory or stigmatising care’ reports on experiences of pejorative and inequitable care.

**Conclusions:**

An equity lens helped identify areas of discriminatory and inequitable care. Key recommendations include cultural safety training for staff; service-user engagement and co-production of research and resources, and appropriate facilities and recording systems to facilitate individualised, needs-based maternity care.

**Supplementary Information:**

The online version contains supplementary material available at 10.1186/s12884-022-05279-6.

## Background

Urgent calls to address systemic injustice continue to intensify across maternal and new-born research, guidance and policies [1]. Women from minoritised ethnic communities face poor maternal and infant health outcomes [2–4]. The ‘Mothers and Babies: Reducing Risk through Audits and Confidential Enquiries’ [MBRRACE] report reviewed maternal deaths in the UK over 2017–2019, and found that in comparison to White women, Black women are more than four times more likely to die from pregnancy- and childbearing-related complications, and women from Asian ethnic backgrounds are almost two times more likely to die [5]. The audit also confirmed minoritised ethnic women are at higher risk of experiencing premature birth, stillbirth or neonatal deaths [5]. A further review of UK maternal mortality between 2009–2017 noted this inequality between Black women and White women was widening [6]. Similar patterns of inequity in mortality and morbidity outcomes for minoritized women are being reported in other countries, such as the United States [7–9], Finland [10] and the Netherlands [11].

These disparities may be partly health-related (e.g., higher risks of diabetes and heart disease) [12], or due to material disadvantages (e.g., living conditions, occupations) and structural inequalities [13]: women from minoritised ethnic communities are more likely to experience poverty, have poorer educational outcomes and higher unemployment [14, 15]. However, minoritised women are also known to have poorer experiences of maternity care [16–19], often receive less antenatal care [5, 20, 21], and face barriers to accessing care [22]. In a UK national cohort study undertaken to explore disparities in maternal mortality rates, multiple areas of bias were identified in the treatment of Black women who died [23]. Health professionals’ use of poor and judgemental communication associated with cultural stereotyping [16, 17, 24–27] and microaggressions [23, 28] have been widely reported. Microaggressions refer to brief, verbal or behavioural and often unconscious interactions that transmit hostility and racial insults [28]. These issues create barriers for women from minoritised communities to make informed decisions, and challenges for healthcare professionals to provide culturally sensitive care [4, 24, 29].

Minoritised ethnic women have been found to have higher rates of poor perinatal mental health, such as antenatal and postnatal depression, when compared to White populations [7–11, 30, 31]. Women who are asylum-seekers or refugees are also more likely to experience mental health problems, including higher rates of depression, Post Traumatic Stress Disorder [PTSD] and other anxiety disorders [32–34]. In some minority ethnic communities, poor mental health is stigmatised and a lack of validation can create barriers to care [35, 36]. As different communities understand mental health in different ways, available information on poor mental health and how to access help may not suit the needs of all communities, meaning that women are less likely to access support [35–37].

The UK Better Births maternity transformation agenda [38] and more recent national policy [39, 40] call for equitable and culturally safe care to address systemic racism, discrimination and healthcare disparities. It is argued that further research into the needs and care of minoritised ethnic perinatal women is needed to understand their needs, to help inform culturally sensitive care and resources, and to inform training to upskill healthcare professionals [29, 41, 42]. As national analysis has limited power to detect which differences in maternity care are significant (Knight 2022), this highlights the importance of research which focuses on the lived experiences of women from minoritised ethnic communities in relation to maternity service provision [26].

The MAINN research unit at the University of Central Lancashire were commissioned by an English North-West Local Maternity and Neonatal System (LMNS) to consult with women from minoritised ethnic communities to explore their experiences of maternity services, including maternity care and mental health support. LMNSs bring together all those involved in providing and organising maternity care in the National Health Service (NHS), such as midwives, obstetricians, service users, neonatal staff, managers, commissioners, public health professionals, educators, perinatal mental health providers and GPs [43]. In response to national directives, in this paper we used an equity lens – defined as the act of being fair and equal (www.merriam-webster.com/dictionary/equity) - to report on key findings that illuminate women’s experiences of inequalities and prejudices, and if and how their cultural and religious needs were met across this service footprint. Recommendations to help reduce inequalities and to improve outcomes are also provided.

## Methodology

### Design and aim

A mixed-methods study was undertaken to explore the maternity care and mental health needs and experiences of perinatal women from minoritised ethnic communities across a LMNS in North-West England. In this paper we adopted an equity lens to report on findings that highlight the extent to which cultural and religious needs were met, and experiences of prejudicial and discriminatory care.

### Data collection

Data collection was conducted between April–August 2021 using online surveys, follow-up interviews, and community consultations.

#### Survey

A survey was developed based on core themes within the NHS Better Births agenda [38], and key literature regarding minoritised ethnic women’s experiences of maternity care [19, 44, 45]. The draft survey was shared with minoritised ethnic women (*n* = 4) and professionals (*n* = 2) working with perinatal women for feedback. While some of the feedback related to survey length or small language changes, other suggestions targeted specific cultural issues, such as help-seeking for mental health issues, and to include other ways in which minoritized women can feel discriminated. Final survey questions explored access to and experiences of antenatal care (including antenatal education); information, communication, and choice; experiences of (dis)respect and judgement; mental health needs and support; cultural/religious needs and support; and overall experiences of maternity care. Survey questions asked respondents to rate their views of statements such as, ‘Overall, could you make yourself understood to the maternity care staff?’, using Likert scales of e.g., 1 (‘always’) – 5 (‘never); with free-text boxes for participants to provide additional explanatory information. Sociodemographic data was recorded. A copy of the survey is available from the lead author.

The survey was hosted on the Qualtrics online survey platform and was available in seven prominent local languages (English, Punjabi, Urdu, Gujarati, Arabic, Polish and Romanian). Four compulsory eligibility statements at the start of the survey confirmed the participant was a) 18+ years, b) had given birth in the last 2 years and c) in the LMNS footprint, and d) from an ethnic minority group. Women who responded ‘no’ to any of these statements were directed to the end of the survey and thanked for their interest. All survey questions were optional, except regarding respondents’ ethnic group; this was so we could ensure that the views of a range of ethnic groups were captured. The survey was distributed via social media and/or email to over 470 contacts including childcare organisations, schools, religious/faith centres, targeted social media forums, and community groups.

#### Interviews

Participants who completed the English-language survey were asked if they would like information about a follow-up interview. Participants could follow a link to provide their contact details; an information sheet regarding the interview was then issued by email. The interview explored all key survey topics, and interviewees were sent a £10.00 e-voucher (if they agreed) after the interview had been completed. The interviews lasted 27–68 minutes and were transcribed and anonymised for analysis purposes.

#### Community consultations

It was recognised that the COVID-19 pandemic could have an impact on data collection (e.g., researchers were unable to access perinatal groups), and that there may be difficulties in collecting information from non-English speaking participants. We therefore invited community leaders/staff (such as diversity leads, staff from religious/faith centres, and perinatal services) to talk to perinatal women on our behalf. Two diversity leads from minoritized backgrounds (employed by other agencies to talk to perinatal women about their maternity experiences) agreed to help collect data for our study. These individuals had no formal background in research and were provided with general instructions to talk to women who met our inclusion criteria and to record basic demographic information and summaries of their responses to key questions (focused on the main topics in the  survey) on a pre-defined Microsoft Form; research participants and diversity leads received E-vouchers to thank them for their involvement.

### Data analysis

Quantitative survey data was uploaded into SPSS for descriptive analysis (%, means). Braun and Clark’s thematic analysis approach [46] was used to analyse the qualitative interviews, survey open text, and consultation data to identify key issues within the data set. The quantitative and qualitative data were then synthesised and merged to creative descriptive themes that represented the whole data set, similar to other studies [47]. Data analysis was led by GT, supported by JC, NC, and MCB; with final interpretations shared with all authors until consensus was agreed. For the purposes of this paper, we adopted an equity lens, defined as the act of being fair and equal (www.merriam-webster.com/dictionary/equity), to specifically focus on data in relation to women’s experiences of inequities and discrimination.

### Reflexivity

All seven authors self-define as female and are from a social science (*n* = 3), nursing (*n* = 1), midwifery (*n* = 1) or health research (*n* = 2) background. Four authors are White British, two are from South Asian backgrounds and one has a Mixed ethnic background. Six of the authors are mothers, and five have a history of research with perinatal women. All authors held pre-understandings of equitable, culturally safe, and respectful maternity care; author triangulation enabled insights into cultural values and beliefs to be considered throughout.

## Findings

This study captured data from 104 service users. There were 91 survey respondents (all of whom completed the English version), and 11 follow-up interviews with 12 women (one interview included both a survey respondent and her wife). Twelve additional women also contributed through the community consultations, undertaken by two diversity leads employed in the locality. Sociodemographic data is reported in Table 1. Most participants self-identified as having an Asian ethnic background (65.0%), followed by Black (10.7%), Mixed (10.7%), White (8.7%) and Other (Arab) (3.9%) ethnic groups. The most common age bracket was 30–34 (32.0%) followed by 25–29 years (23.3%). Most women were primiparous (*n* = 39, 42.9%), just over a third of the sample had a child aged 1–6 months (34.1%), and 27 (25.5%) had a child aged 12–24 months. Two participants (2.2%) had a baby younger than 1 month. Sixteen of the survey respondents (17.6%) were born outside the UK and from those who provided details, most (*n* = 11/14, 78.6%) had been in the country for over 5 years.

**Table 1:** Sociodemographic details of participants

**Ethnicity**		
Asian	All Asian backgrounds	67 (65.0%)
	Asian or Asian British – Indian	44 (42.7%)
	Asian or Asian British – Pakistani	15 (14.6%)
	Asian or Asian British – Bangladesh	3 (2.9%)
	Chinese	1 (0.9%)
	Other Asian background	4^b^ (3.9%)
Black	All Black backgrounds	11 (10.7%)
	Black or Black British – Caribbean	2 (1.9%)
	Black or Black British – African	6 (5.8%)
	Black ethnicity	3^b^ (2.9%)
Mixed	All Mixed backgrounds	11 (10.7%)
	Mixed Black Caribbean and White	6 (5.8%)
	Mixed Asian and White	4 (3.9%)
	Other Mixed background	1 (0.9%)
White	All White backgrounds	9 (8.7%)
	White-British, English, Northern Irish, Scottish, Welsh^a^	2 (1.9%)
	White-Irish	1 (0.9%)
	Other White background	6 (5.8%)
Other	Arab	4 (3.9%)
Missing data		2 (1.8%)
**Age**	20–24	7 (6.8%)
	25–29	24 (23.3%)
	30–34	33 (32.0%)
	35–39	22 (21.4%)
	40–44	12 (11.6%)
	45–50	2 (1.9%)
	Missing data	3 (2.9%)
**Number of pregnancies**^c,d^	1	37 (40.7%)
	2	17 (18.7%)
	3	19 (20.9%)
	4	7 (7.7%)
	5+	10 (11.0%)
	Missing data	1 (1.1%)
**Number of children**^c^	1	39 (42.9%)
	2	25 (27.5%)
	3	17 (18.7%)
	4	5 (5.5%)
	5+	2 (2.2%)
	Missing data	3 (3.3%)
**Youngest baby’s age (months)**^c,e^	<1 month	2 (2.2%)
	1–6 months	31 (34.1%)
	7–12 months	19 (20.9%)
	13–18 months	12 (13.2%)
	19–24 months	13 (14.3%)
	25–29 months*	12 (13.2%)
	Missing data/not recorded	2 (2.2%)
**Annual household income**^c^	Below £10,000	7 (7.7%)
	£10,000-17,640	11 (12.1%)
	£17,640–£30,000	13 (14.3%)
	£30,000–£40,000	16 (17.6%)
	Above £40,000	26 (28.6%)
	Don’t know	4 (4.4%)
	Prefer not to say	12 (13.2%)
	Missing data	2 (2.2%)

^a^One respondent identifying as White British appeared to belong to a religious minority as they reported religious needs in subsequent sections of the survey, and one respondent identifying as White British had been born outside the UK

^b^Some of the ethnicity data from the community consultations was recorded in more general classifications such as ‘Asian’ or ‘Black ethnicity’

^c^Collected for survey participants only

^d^ Twenty-two (24.2%) participants reported more pregnancies than number of children, which could relate to a perinatal loss, or a twin/multiple birth

^e^ Thirty-four (37.4%) gave birth to their youngest baby before March 2020 (i.e., prior to the COVID-19 pandemic). For the remainder, it is likely their perinatal care was affected due to the pandemic

Annual household income ranged from seven (7.7%) participants who reported an annual income of below £10,000 to 26 (28.6%) whose annual household income was above £40,000. Compared to national data [48] a similar percentage of families had an income level below £10 K (7.7% v. 6%), but a lower percentage of participants in our sample had a household income above £40,000, compared to the UK as a whole (28.6% v. 40%).

Sixteen survey participants (17.6%) reported a current mental health diagnosis. Nine (56.3%) received their diagnosis before they had their youngest baby, and seven (43.7%) after the birth of their youngest baby.

An overview of all the descriptive level data from the whole study is provided in Supplementary File 1. Overall, our findings resonate with issues reported elsewhere, both for minoritised ethnic women [4, 5], and from general populations [49]; while most participants provided positive feedback for all aspects of their maternity care, a percentage (~20–30% in our study) highlight negative aspects. In this paper we report on survey responses and qualitative insights within four key themes (‘Accessing care’, ‘Communication needs and resources’, ‘Meeting cultural needs’ and ‘Discriminatory or stigmatising care’) to help explain why and how minoritised ethnic perinatal women do not receive equitable care.

### Accessing care

In this theme we report on data related to access to antenatal care and antenatal education. These factors are linked to outcomes and therefore are significant when considering equity in service provision [6]. The 2021 UK guidance [50] recommends that women should see a midwife by 10 weeks gestation, recommending 10 routine appointments for nulliparous and seven for parous women. While almost all participants (*n* = 85, 93.4%) stated that they understood the importance of antenatal care, 74.7% (*n* = 68) of the survey participants saw a midwife in the first 12 weeks of pregnancy; thereby indicating that just over a quarter of women accessed late. Regarding the number of routine appointments, 64.1% (*n* = 25/39) of participants expecting their first baby reported nine or more antenatal contacts, but 30.8% (*n* = 12/39) had eight or fewer. Of participants with older children, 29/49 (59.2%) had nine or more antenatal contacts; however, 14/49 (28.6%) had 5–8 antenatal contacts, and 4/49 (8.2%) had fewer than four.

While over half of participants (*n* = 53; 58.2%) found it ‘easy’ or ‘very easy’ to attend antenatal appointments, 14 (15.4%) found it ‘quite’ or ‘very’ difficult, with some reflecting on the time and cost challenges associated with lengthy commutes to hospital:

Community midwife was easy to access. However, I often had to travel further away for scans and consultations which was expensive, tiring and impacted my job. (Survey respondent #36)

Some women ‘dropped’ attendance at antenatal care due to it not being ‘very robust’, or not ‘important’ (Survey respondent #4). Others complained that they ‘didn’t really get a lot of stuff from the community midwife or the antenatal staff’ (Interview #14), with some reporting a perceived difference between what was provided for first-time and more experienced mothers, with multiparous mothers feeling ‘on your own’.

Only 21 (23.1%) participants had attended antenatal education. More participants expecting their first baby attended antenatal classes (*n* =15/39, 38.5%) compared to only four of 49 participants with older children (8.2%). Further analysis was undertaken to see whether low access was related to the COVID-19 pandemic. Our data revealed that only 20.0% (*n* = 12/60) of women with a baby born in 2020 or 2021 attended antenatal education compared to 31.0% (*n* = 9/23) with a baby born in 2019. While there is no national data on access to antenatal education to draw comparisons between different ethnic groups, the low numbers of women accessing are a concern. Some qualitative comments help to understand low attendance, with comments that; ‘information wasn’t consistent either around antenatal classes and stuff’ (Interview #14) and complaints about staff non-attendance: ‘We went to our first and the room was full and the healthcare professionals or whoever who were due to deliver [the class] just didn’t turn up’ (Interview #3). A lack of, or confusing information also led to several women paying for antenatal education, thereby increasing inequities:

I was told there were no antenatal classes so paid for NCT [National Childbirth Trust] when local Children’s Centre was running them for free. (Survey respondent #20)

### Communication needs and resources

In this theme we report views on whether the maternity systems provided adaptations and resources to meet women’s specific communication needs. Overall, only one survey respondent, and some of those who participated in the community consultations, reported feeling unable to comfortably communicate in or read English. Some of these individuals referred to being offered information and resources (such as a translation service) in an appropriate language during their care. Interpreters were reported to facilitate good care and information exchange:

Lots of staff were around to help and [I] had a translator that [helped me so that I] understood all the recommendations. (Community consultation #4)

Others reflected on how an absence of translators when ‘trying to get others to understand what you’re saying would be ‘quite traumatic’ (Interview #6). One interviewee reported filling this gap for family members, while recognising the limitations:

I end up doing a lot of translating for or explaining things to [relative]. She doesn’t understand. So, if somebody's speaking to her in English, I don't think she gets what they're saying…she'd come home and then she’ll call me and say, “Oh, what's this? What's that?” And then I read up on it and I would tell her, whatever she needs to know. So, she had no opportunity to ask questions, she's not really understood what they're saying, so she can't really ask them anything. She just nods her head and goes away, but that meeting's important, with the doctor or the nurse or the midwife. (Interview #18)

Only 20 (22.0%) participants said they were offered any culturally appropriate information or resources, with 57 (62.6%) answering ‘no’ to this question (although likely related to most of our participants being able to communicate in English). The resources highlighted by participants included translators, as well as translated leaflets, posters and information on topics such as mental health, vaccinations, ultrasound scans, ‘breastfeeding and fasting in Ramadan’, and infant care. However, despite these positive reflections, gaps in obtaining translated material for certain languages, such as Polish, were highlighted. A lack of translated information for use during labour, coupled with a delay in available translation services, was also noted:

And I did notice there wasn't an easy translation service, or any leaflets or information translated into different ones that were readily available when you were in the middle of labour. So, they were relying on like translations over a phone with poor signal. Maybe it’s different in non-COVID times, but right now when you're in the middle of labour, you haven't got time to wait 20 minutes for someone to call you back with a translator. (Interview #14)

One woman referred to the need for condition-specific information (such as hyperemesis gravidarum, postnatal depression) due to a lack of recognition and understanding of these issues within some minoritised communities:

And people that don't speak English as their first language or who live with in-laws who don't have supportive husbands. I think they’re women who would really struggle. Because in the [ethnicity] community, the [ethnicity] culture they don't understand postnatal depression. They don't understand hyperemesis. “It didn't happen to us, so why is it happening to you? Oh, you're just exaggerating. Oh, you're really soft.” I had to put a brave face on it because I was sick, and it just hadn't happened to them, and they couldn’t understand why I was getting it so bad … If they're not speaking in English, then I think there's need, people out there that are going to give them that information, and that support, you know, “Hey, this is out there”. They wouldn’t know. (Interview #18)

Women also raised issues about the taboo nature of mental health. Overall, 42 survey respondents felt their mental health was negatively affected by the birth; twenty-five (59.5%) felt able to talk about these feelings and 17 (40.5%) did not. UK guidance stipulates that all women should be asked about their mental health at every appointment with a maternity care professional [51], yet over a quarter of survey respondents (*n* = 25, 27.5%) reported not being asked about how they were feeling post-birth. Those who did talk about their feelings were most likely to discuss their mental health with family (*n* = 22, 88%) or friends (*n* = 16, 64%), compared to midwives (*n* = 7, 28%), doctors (*n* = 2, 8%) and support organisations (*n* = 4, 16%). While this suggests that women may prefer communicating their mental health issues within their personal networks, several women raised issues about sharing their concerns with healthcare providers. Some suggested that women would not always give an honest response: ‘I think generally people would just say they’re fine’ (Interview #18) which may be due to women feeling ‘embarrassed’ and how poor mental health ‘isn’t seen as a problem in the Asian community’ (Survey respondent #33).

### Meeting religious and cultural needs

In this theme we report data that concerns whether the support met women’s specific religious and cultural needs. While some women reported that they ‘didn’t have any religious or cultural needs’ (Survey respondent #26), almost a third of the survey respondents (*n* = 27, 29.7%) felt their religious and/or cultural needs were only met ‘to some extent’ or ‘not at all’. The extent to which women’s different needs were met by maternity professionals are detailed as follows:

#### Seeing themselves reflected in the services

Some women said it was becoming common to see more inclusive images, within the antenatal service and elsewhere:

I do feel like certain images, they are mainly of White women. But recently I think after I’ve given birth and I've seen, I don't know, it might be a bit in the neonatal unit, but there's a lot of images of like hijab girls and hijab nurses. But before you wouldn't see that … You would only see White nurses; you wouldn't see an Asian nurse. (Interview #5)

Another woman reflected on how relatable images helped to normalise her personal practices:

But just seeing [on a social media post] another woman with a headscarf. It just normalizes it. I see it and I think “Oh it is ok to wear a headscarf if you wanted to”. (Interview #6)

#### Female care provider

Overall, 63 participants (69.2%) indicated that having a female care provider was important to them. Of these, only 26 (41.3%) were offered a female care provider. Twenty-four (26.4%) respondents considered there had been times (one said, ‘always’) when their privacy was not upheld; on most occasions this concerned the presence of male staff when women were not ‘appropriately’ covered.

I'm a Muslim. And there's certain things that, you know, we have like limits and boundaries, and certain body parts that we need to cover in front of the male. (Interview #5)

Some Muslim interview participants said they felt comfortable asking for a female provider, but their requests were not met. One had requested a female sonographer due to her ‘religious’ values, but this had been overlooked, ‘and it was a male colleague there’ (Interview #10). A participant in the community consultations discussed how her request for no male healthcare professional was not acted upon:

She didn't want any man in the day of labour to be with her in the room from her religion background and she put that note in her maternity notes, but on the day of labour there is a man there. (Community consultation #9)

Some women referred to maternity staff asking whether a male healthcare professional would be acceptable, while others reported a lack of choice. One woman described her perceived inability to wear her headscarf during a caesarean section:

I wear a head scarf. So, the surgeons were male, and I almost felt a little bit uncomfortable about that. Well, I don't really expose any part of my body. And yeah, in that situation, I mean, I understand when you’re on the table, but when you're moving around, there are other people around and your legs are showing, your body is showing, your hair is showing. That I found a little bit uncomfortable…I thought perhaps they don't want me to wear a headscarf, so I didn't say anything because we were in the midst of the pandemic. (Interview #18)

The request for a female provider was not a refusal to be treated by men, and participants were clear that the requirement is different in an emergency medical situation:

I never had to ask because I think there was only one scan and that was a scan that happened in emergency. That was done by a male sonographer, but at that point from a religious belief perspective as well, it was an essential care situation, and I had my sister-in-law with me. (Interview #3)

#### Prayer opportunities and facilities

Some Muslim women discussed their practice of a prayer being recited into the new-born baby’s ear (the ‘call to prayer/Adhan’). One woman noted that her care providers had accommodated this, but she felt more awareness would have been helpful:

I did do a birth plan and in there it said certain things like to have some space afterwards where my husband could recite a prayer in the new-born baby’s ear. That’s how we sort of welcome a new baby. It's usually the dad who whispers a prayer into the baby’s ear. And so just, I think more than that, the staff members aren’t awareof what's going on, they might be alarmed. But it was just to say, look, this is what this is what we do. And they were brilliant. They really were accommodating of everything. (Interview #10)

Another woman referred to how this request was ignored:

Putting Britney Spears songs on in theatre when my birth plan explicitly stated that I wanted my husband to be praying our religious call to the baby. (Survey respondent #28)

Some women made critical comments about the lack of ‘prayer facilities’ in maternity units, as well as a lack of appropriate washing facilities:

The midwife had said, because my legs and everything were covered in blood, that you can have a bath, because they don't have showers, but the thought of sitting in a bath. Which would then be filled with my blood. Just felt so wrong and from a cultural point of view, if they were actually looking at my religion and my culture, we wash in running water. We don't wash in stagnant water. So, it's a relaxation or for a soak for ailments. Not to wash anything because you're not washing it. You’re sitting in it. Yeah, I just wanted a stand-up shower. And they said, “Well we've got a bath”. (Interview #19)

#### Placenta

The need to take the placenta home to bury it arose in several interviews with Muslim women, revealing a range of experiences and attitudes. Some women found this practice was integrated into their maternity care:

We have to bury the placenta and I put that in our birth plan. After I gave birth the two midwives who were there, as I said “Oh I need to…”, one of the midwives said, “Yeah we just need to check it”, before I even said, obviously that I need to take it. That to me, it kind of showed that understanding that they know that I would like to take it home because I’m Muslim. So, they packed it all in the medi-bag for us. (Interview #6).

Others found that they were unable to follow this part of their practice, despite asking:

And then as part of our religious practices, we take the placenta, and we bury it. And they didn't ask me. So, when the consultant came and I said, “Do you know what's happened to the placenta?”, they said, “Oh, I think they've sent it for lab testing”. So, I said, “Well can I get it back?”. And they said, “I don't know, I'll try to find out”. And nobody ever got back to me about that I didn't receive that so, and I asked a couple of times, but I guess then it just became such a frustration that I'd been let down. I didn't pursue it. (Interview #7)

Some women said this practice was not necessarily religious, but rather cultural; ‘Islamicly, we should bury that? But it’s not a problem. It’s not a huge problem if you don’t, it’s not a sin’ (Interview #18). Other interview participants said they had never heard of this practice before we asked them, which underlines the heterogeneity of the study population.

#### Dietary/vegetarian options

Just under half the participants (*n* = 45, 49.5%) had been an inpatient in hospital while pregnant or having their baby. Of these, 23 (25.3%) felt their dietary needs had only been met ‘to some extent’, or not met at all. Qualitative comments provided polarised feedback. Some participants praised being given choices of ‘halal’ or ‘good suitable vegetarian options’. Others complained of restricted choices, such as ‘no halal options’, ‘the vegetarian options were just salads’, and gave negative feedback on the quality of food; ‘it was bland and boring’, ‘the food was horrible’ (e.g. Survey respondents #14,#68, #15). One woman spoke about how staff made dietary (religious) assumptions based on her country of origin:

But then they always ask you, like, are you OK with meat? You know, the different kinds of meat. We thought you’re a vegetarian, because they think because I’m [nationality], everybody's a vegetarian. (Interview #9)

An associated dietary issue related to a lack of information when trying to establish whether the ‘Vitamin K vaccination was vegetarian or not’ (Survey respondent #30).

### Discriminatory or stigmatising care

In this theme we focus on women’s experiences of discriminatory or stigmatising care from maternity professionals. Just over half (*n* = 49, 53.8%) the participants ‘never’ felt that maternity care staff were less positive or caring towards them, compared to other women, reflected in comments such as ‘I personally was treated brilliantly’ (Survey respondent #71), and ‘the health visitor respected our needs and didn’t make me feel different for being Pakistani’ (Survey respondent #62). However, just over a third (*n* = 31, 34.0%) of survey participants felt they were treated differently to other women.

Numerous women felt they were treated differently based on their ethnic background. One stated: ‘other women in my room who were White were looked after more and attended to and offered more help’ (Survey respondent #82). Another noted how a receptionist at the antenatal clinic displayed microaggressions by ‘speaking loudly’ and was ‘short and rude to anyone who didn’t speak English as a first language’ (Survey respondent #66). Another mother reported:

Many of the staff especially after my delivery treated me like I was thick. They spoke to me loudly and slowly assuming I didn't know English and were quite vicious in manhandling my breasts to feed my child even though my milk hadn't come through. They didn't allow me to hold my sleeping child during the hospital stay as they said it would cause death by suffocation. They were ill-informed, racist, and rude. (Survey respondent #28)

One participant described a series of interactions with a maternity care provider which she described as ‘racism’; ‘it was the racism thing that got to me more than anything else’. The first occasion occurred during booking when the midwife recorded her ethnicity as ‘Asian’ rather than ‘mixed race’. When she told the midwife ‘“I’m mixed race, I classify myself as [ethnicity] and [ethnicity]”’, the midwife disagreed, stating, ‘“you’re medically Asian”’. The woman was then left wondering, ‘will it matter in the rest of my notes?’. The second occasion occurred during the first postnatal community visit when the same midwife dismissed the mother’s concerns about jaundice based on the baby’s (presumed) skin colour, without having seen the infant:

I just mentioned that I think [baby’s] a bit jaundiced. And she said, “Well, with your skin tone [baby’s] going to appear a bit yellow”. And then I brought [baby] down and [baby] was jaundiced. So, I just thought, that's not the first thing that you say to someone if they are concerned about jaundice, it was just because I'm [ethnicity]. It's a myth that they're yellow, they're not, you’re not yellow-skinned, they’re [ethnicity]. Not like jaundice yellow. I’m not that stupid. (Interview #14)

A few of the women also struggled with whether their lack of non-consensual and damaging care (e.g., sustaining an injury during an internal examination) was due to other factors. For one, this was whether it was because she had tested positive for COVID-19, or was related to her ethnic minority status:

I don't know the aftercare, was it because of my ethnic minority status? I just feel like I was treated differently. And I even said to the staff that “I feel like a leper because of my COVID status”. But whether it's my COVID status with my ethnic minority, I was treated differently. (Interview #7)

Just over a third of survey respondents felt that maternity care staff made incorrect assumptions about them (*n* = 29, 31.9%), which on occasion were associated with cultural biases. On some occasions, assumptions were made about women’s sexual orientation, with inappropriate references being made to the father of the baby, ‘despite me being in a same-sex relationship’ (Survey respondent #21). One woman referred to a doctor speaking in a ‘really offensive way’ when she asked a medical question and felt he had assumed ‘I have no education’. When she told him her job, the doctor commented ‘“nowadays women can go to a university”’ (Survey respondent #28); the woman subsequently made a complaint. She referred to how ‘healthcare staff all need continuous and up to date training’ on how to talk with women from different ethnic minorities; ‘I am an educated university lecturer, yet they treated me like I was illiterate from a foreign country leaching off benefits in the UK’ (Survey respondent #28).

The need for more cultural awareness concerning Muslim women’s social circumstances, i.e., being a single parent, was also highlighted by participants. This situation can lead to women being ostracised, and is associated with stigma, shame, and poor mental health:

I think a little bit of cultural awareness would have helped…yeah I said to her that you know, it’s not OK, it’s not normal within the community [single motherhood], so I know I’ve got everything stacked against me….So she [midwife] was aware of it, but I don’t think she was aware of the impact or the severity of it…it was a further barrier for me and gave me more pressure, which would then affect my mental state as well as aftercare for my baby and stuff. (Interview #1)

Despite her disclosures, this woman felt she was offered ‘no attention to detail, there was no mental health care’. The only acknowledgement of her marital status was from a doctor who asked, ‘“how are you going to deal with your problem socially?”’. She felt the health visitor ‘didn’t pick up on anything either’ despite describing her ‘anxiety’, ‘suspicious thoughts’ and ‘paranoia’. Eventually she was admitted as a voluntary psychiatric patient, but as no mother and baby places were available, she was separated from her new baby:

So, I had to go through a massive breakdown and then be admitted into hospital without being with my baby in a section ward without being sectioned myself - it was just horrific….that had a massive impact. I had a whole week away from my child after believing something terrible was going to happen to [baby] and how [baby] was going to be taken away from me. Because my nightmare literally did happen. (Interview #1)

Overall, only 59 (64.8%) survey respondents felt they were ‘always’ able to have confidential discussions with maternity care staff; 27.5% reported this had only happened ‘sometimes’, ‘not often’ or ‘never’. One woman referred to how her ‘confidential’ pregnancy status was breached by a midwifery student from the same ethnic background, and how this could have caused her to be stigmatized within her community:

My pregnancy was confidential for my own safety. All staff respected that. However, after a few months I found out one of the students on placement had told friends of mine she seen me on the ward post-birth. This could have caused a real big problem for me. This could have been a great danger to myself and my mental health. She was also [ethnicity] same as me which is why she is more likely to do this. I never complained as I didn’t want anyone to get in trouble. (Survey respondent #48)

## Discussion

The findings reported here are part of a larger study to explore minoritised ethnic perinatal women’s experiences of maternity services, including maternity care and mental health support. In this paper we specifically adopted an equity lens to focus on whether women’s cultural and/or religious needs were met and to identify areas where discriminatory and inequitable care was experienced. Overall, ~20–30% of our study sample considered that their cultural and religious needs were not understood or supported by maternity care providers; with some experiencing what they considered to be racist actions and behaviours. Exposure to interpersonal racial discrimination may increase the risk of poor perinatal outcomes [5, 52], and maternal experiences of racism are associated with poorer outcomes for children [53].

A lack of early pregnancy contact can contribute to a poor midwife-woman relationship [54], and complications [6]. In our study, over a quarter of survey participants did not see a midwife in the first 12 weeks of pregnancy. We also found a sizeable number of women did not access antenatal education, with the percentage of women attending being higher during COVID compared to previously. In the wider literature it is reported that ‘late bookers’ are typically from marginalised groups, including minoritised ethnic communities [55]. A qualitative study, which included women from minoritised ethnic backgrounds, explored reasons for late access; key reasons concerned avoidance due to ambivalence, fear, not valuing self-care, and professional/system failures [56]. Moreover, some of the barriers we found in relation to non-access to antenatal education amongst minoritised ethnic women are also reported by Tighe [57] in terms of ambivalence, a lack of information and transportation difficulties.

A recent systematic review identified UK maternity services and systems as having a dominant residual effect on communication, midwife-woman relationships, and cultural and social needs relating to ethnic health inequalities [4]. Staff shortages and time constraints by maternity staff can mean that women’s expectations of support are often not met, leading to feelings of isolation, especially among those with mental health needs [4, 17, 58]. However, notwithstanding service constraints, our results echo other recent findings of multiple areas of bias in maternity care, including lack of nuanced care and microaggressions [23, 59]. Some women in our study reported prejudice or discrimination based on their ethnicity, religion or culture [54] and being treated in an unsympathetic way, especially compared to the ways White mothers were seen to be treated. These insights are familiar to other studies from the UK literature on maternity care, which reports direct discrimination, stereotyping or racist comments in a range of geographical settings [17, 18, 29, 60–62]. It is also argued that these negative practices can reinforce implicit biases of expecting minoritised ethnic women to adapt to an insensitive and sometimes discriminatory health system, rather than the system being responsive to the needs of its users [63]. These insights highlight the need for cultural safety training amongst health care staff to raise awareness of unconscious biases and increase understanding of specific religious and cultural needs; similar work has been undertaken in providing culturally safe maternity care in other countries, such as Aboriginal and Torres Strait Islander families in Australia [64], and indigenous mothers in Canada [65]. Our findings also indicate that some ethnic minority women would rather seek help for mental health problems from family members and friends than from a healthcare provider [35, 66]. These insights support other studies which found this may be a reactive or protective response; staff attitudes and behaviours may inhibit women seeking help from services, and when disclosure does take place, it may not be met with a supportive response [67, 68].

As reported elsewhere, women in our study who were not confident in English often reported language barriers and interpretation challenges when trying to communicate with maternity staff [17, 19]. The need for unrestricted access to interpretation and translation services has been identified by midwives as essential for the provision of effective, holistic maternity care [63, 69]. But a recent UK study indicated that staff reactions to non-standard accented English can also impact women’s engagement with services [26]. Higginbottom and colleagues found that ethnic minority women with positive perceptions of maternity services valued open communication with health professionals in meeting their needs, and this was reflected in our data [45]. This links to the significance of continuity of care by an appropriate team [38], which was deeply appreciated by our participants when it occurred. However, this is ‘rarely evident’ for ethnically minoritized women [23], and the lack of communication and consistency of information inherent in a lack of continuity of care can lead to dissolution of trust [26].

In February 2021 the UK government announced a Maternity Disparities Taskforce to tackle inequalities in maternity care, together with supporting guidance [39]. Its focus includes improving personalised care and support plans, addressing how wider societal issues affect maternal health, increasing access to maternity care for all women and developing targeted support for those from the most vulnerable groups and empowering women to make evidence-based decisions about their care. While this is a positive move to help address inequalities and to improve maternity care outcomes for these populations, it is important to contextualise women from minoritised ethnic backgrounds as a diverse population, with different individual, community or group needs [4]. Through our work we were able to identify several recommendations that can complement the national and international priorities to improve the care of these women (see Table 2). The recommendations do not suggest a ‘one size fits all’ approach but raise awareness of issues that some women can face and provide potential solutions.

**Table 2:** Recommendations to improve the care and support of minoritised ethnic women

Area	Recommendations
*Cultural safety training*	Cultural safety training is needed for maternity care staff to raise awareness of unconscious biases in terms of language and interpersonal behaviours that women experience as potentially ‘*discriminatory*’ and ‘*racist*’, and to increase understanding of specific cultural and religious needs, including: o women may need their body and hair to be covered during care o parents may have requests for prayers at time of birth o parents may want to take their placenta home o parents may want vegetarian options in vitamin K vaccinations o the challenging nature of mental health or other social issues within specific family-based situations (i.e., single parenthood)
*Developing appropriate resources*	Services need to consult with the women they serve to understand the needs of their local population, and co-design services accordingly. This should include co-creating: o Content (written, or digital) on suitable topics, including screening and identification of mental health issues o Information to be translated into appropriate languages o Appropriate signage to ensure women feel included
*Recording systems*	o Systems need to be adapted to ensure women’s self-disclosed ethnicity can be recorded (rather than fit pre-defined classifications) o Maternity staff should proactively ask about, document, and routinely consult with parents about cultural or religious needs at initial and subsequent appointments (such as via birth plans)
*Dietary options*	o More suitable menu options are required to meet dietary needs
*Female healthcare provider*	o Staff need to acknowledge this preference and ensure this can be provided wherever possible
*Facilities*	o Suitable prayer and washing facilities should be offered in delivery and birth centre settings.

### Strengths and limitations

The remit of this study was focused on a very targeted population. We were aware that we were addressing a sensitive issue, with mental health being a taboo subject in certain ethnic groups and we reached out to over 470 contacts to help with recruitment. Therefore, while these insights are unlikely to offer a complete representation of women’s views in the locality, our report on the experiences of 100+ women are a key achievement.

A key limitation was undertaking this project during the COVID-19 pandemic when key forums for data collection, such as perinatal groups, were closed. While efforts were made to include women of different languages, none of the translated surveys were completed, despite targeted recruitment and dissemination. This raises questions of cultural sensitivity, digital access, and literacy. However, it may reflect others’ insights that stereotyping can make maternity service-users feel excluded, and negatively impact on women’s ability to speak up about their experiences [13, 70]. While further work to ensure the cross-cultural validity of the research questions may be helpful [71], innovative approaches to capture the views of these women are also needed. While the community consultations provided an important and valuable recruitment strategy to engage women in their own languages, the quality and depth of the data was limited. Future use of this approach should therefore incorporate training and ongoing support, to maximise these opportunities and build capacity.

## Conclusion

In this study we adopted an equity lens to focus on the extent to which minoritised ethnic perinatal women’s cultural and/or religious needs are met within maternity services, and to identify key areas where discriminatory and inequitable care is experienced. The findings describe direct experiences of discrimination, racism, and marginalisation, that can help explain higher levels of dissatisfaction, reticence in help-seeking, and poor mental health in this population. These insights help to inform and reinforce national as well as international directives to improve service provision and outcomes for minoritised ethnic women and their families. While further research is needed to assess the impact and experiences of solutions to optimise individual and needs-led care, key recommendations identified include: the need for cultural safety training for healthcare staff; service-user engagement and coproduction to develop suitable resources; recording systems to detail self-disclosed ethnicity, and appropriate facilities and support that are aligned with religious and cultural needs.

## Supplementary Information

**Additional file 1: Supplementary File 1.** Summary of all survey responses. **Table 1.** Antenatal care. **Table 2.** Experiences of care and care needs.

## Data Availability

All key data generated or analysed during this study are included in this published article [and its supplementary information file].
