# Supplementary material for: Minoritised ethnic women’s experiences of inequities and discrimination in maternity services in North-West England: a mixed-methods study
Source: BMC Pregnancy Childbirth. 2022 Dec 22;22:958. doi: 10.1186/s12884-022-05279-6 (PMC9773462; doi:10.1186/s12884-022-05279-6)
Supplement: Supplementary file 1 — Additional file 1: Supplementary File 1. Summary of all survey responses. Table 1. Antenatal care. Table 2. Experiences of care and care needs. [file 12884_2022_5279_MOESM1_ESM.docx]

**Supplementary File 1 – Summary of all survey responses**

**Table 1 - Antenatal care**

|  | | N (%) | | |
| --- | --- | --- | --- | --- |
|  | | All participants (n=91) | Participants expecting first baby (n=39) | Participants with older children (n=49) |
| *How many weeks pregnant were you when you first saw a midwife?* | Under 8 weeks | 14 (15.4%) | 8 (20.5%) | 5 (10.2%) |
|  | 8-12 weeks | 54 (59.3%) | 20 (51.3%) | 33 (67.3%) |
|  | 13-16 weeks | 11 (12.1%) | 5 (12.8%) | 6 (12.2%) |
|  | 17-20 weeks | 2 (2.2%) | 1 (2.6%) | 1 (2.0%) |
|  | Over 20 weeks | 6 (6.6%) | 3 (7.7%) | 2 (4.1%) |
|  | Missing data | 4 (4.4%) | 2 (5.1%) | 2 (4.1%) |
| *Participants born outside the UK (n=16)* | Under 8 weeks | 5 (31.3%) | – | – |
|  | 8-12 weeks | 5 (31.3%) | – | – |
|  | 13-16 weeks | 3 (18.8%) | – |  |
|  | Over 20 weeks | 1 (6.3%) | – | – |
|  | Missing data | 2 (12.5%) | – | – |
| *How many times did you see or speak to a maternity care professional when you were pregnant? (in person, telephone, or video call)* | 0-4 times | 10 (11.0%) | 6 (15.4%) | 4 (8.2%) |
|  | 5-8 times | 21 (23.1%) | 6 (15.4%) | 14 (28.6%) |
|  | 9-11 times | 25 (27.5%) | 11 (28.2%) | 14 (28.6%) |
|  | Over 11 times | 31 (34.1%) | 14 (35.9%) | 15 (30.6%) |
|  | Missing data | 4 (4.4%) | 2 (5.1%) | 2 (4.1%) |
| *How easy was it to access antenatal care?* | Very Easy | 22 (24.2%) | 10 (25.6%) | 10 (20.4%) |
|  | Easy | 31 (34.1%) | 14 (35.9%) | 17 (34.7%) |
|  | OK | 20 (22.0%) | 9 (23.1%) | 11 (22.4%) |
|  | Quite difficult | 12 (13.2%) | 4 (10.3%) | 8 (16.3%) |
|  | Very difficult | 2 (2.2%) | 0 | 1 (2.0%) |
|  | Missing data | 4 (4.4%) | 2 (5.1%) | 2 (4.1%) |
| *Did you understand why it was important to have antenatal/pregnancy care?* | Yes, fully | 76 (83.5%) | 30 (76.9%) | 44 (89.8%) |
|  | Yes, to some extent | 9 (9.9%) | 7 (17.9%) | 2 (4.1%) |
|  | No, not at all | 1 (1.1%) | 0 (0%) | 1 (2.0%) |
|  | Missing data | 5 (5.5%) | 2 (5.1%) | 2 (4.1%) |
| *Did you attend antenatal education classes?* | Yes | 21 (23.1%) | 15 (38.5%) | 4 (8.2%) |
|  | No | 65 (71.4%) | 22 (56.4%) | 43 (87.8%) |
|  | Missing data | 5 (5.5%) | 2 (5.1%) | 2 (4.1%) |

**Table 2 – Experiences of care and care needs**

|  | | N (%) |
| --- | --- | --- |
| **Information giving** | | |
| *Overall, did you understand the information or advice that the maternity care staff gave you?* | Always | 53 (58.2%) |
|  | Most of the time | 25 (27.5%) |
|  | Sometimes | 4 (4.4%) |
|  | Not often | 2 (2.2%) |
|  | Never | 0 |
|  | Missing data | 7 (7.7%) |
| *Were you able to have confidential discussions with the maternity care staff?* | Always | 59 (64.8%) |
|  | Most of the time | 14 (15.4%) |
|  | Sometimes | 6 (6.6%) |
|  | Not often | 4 (4.4%) |
|  | Never | 1 (1.1%) |
|  | Missing data | 7 (7.7%) |
| **Being heard by maternity care staff** | | |
| *Overall, did you feel that the maternity care staff listened to you?* | Always | 38 (41.8%) |
|  | Most of the time | 32 (35.2%) |
|  | Sometimes | 8 (8.8%) |
|  | Not often | 4 (4.4%) |
|  | Missing data | 9 (9.9%) |
| *Overall, could you make yourself understood to the maternity care staff?* | Always | 60 (65.9%) |
|  | Most of the time | 18 (19.8%) |
|  | Sometimes | 4 (4.4%) |
|  | Not often | 2 (2.2%) |
|  | Missing data | 7 (7.7%) |
| **Choice and decision-making in maternity care** | | |
| Did *you feel that you were able to have a choice about what happened to you during your maternity care and childbirth?* | Always | 35 (38.5%) |
|  | Most of the time | 20 (22.0%) |
|  | Sometimes | 14 (15.4%) |
|  | Not often | 10 (11.0%0 |
|  | Never | 3 (3.3%) |
|  | Missing data | 9 (9.9%) |
| *Did you feel involved in the decisions about what happened to you during your maternity care and childbirth?* | Always | 40 (44.0%) |
|  | Most of the time | 22 (24.2%) |
|  | Sometimes | 6 (6.6%) |
|  | Not often | 11 (12.1%) |
|  | Never | 3 (3.3%) |
|  | Missing data | 9 (9.9%) |
| **Explanations and consent** | | |
| *Did the maternity care staff explain any procedures or clinical checks that they recommended?* | Always | 52 (57.1%) |
|  | Most of the time | 19 (20.9%) |
|  | Sometimes | 7 (7.7%) |
|  | Not often | 4 (4.4%) |
|  | Missing data | 9 (9.9%) |
| *Did you understand why you needed to have any procedures or clinical checks?* | Always | 57 (62.6%) |
|  | Most of the time | 16 (17.6%) |
|  | Sometimes | 7 (7.7%) |
|  | Not often | 1 (1.1%) |
|  | Never | 1 (1.1%) |
|  | Missing data | 9 (9.9%) |
| *Did you understand why your baby needed to have any procedures or clinical checks (e.g. after your baby was born)?* | Always | 59 (64.8%) |
|  | Most of the time | 11 (12.1%) |
|  | Sometimes | 8 (8.8%) |
|  | Not often | 3 (3.3%) |
|  | Never | 1 (1.1%) |
|  | Missing data | 9 (9.9%) |
| *Did the maternity care staff ask for your consent before they carried out procedures or examinations?* | Always | 71 (78.0%) |
|  | Most of the time | 6 (6.6%) |
|  | Sometimes | 2 (2.2%) |
|  | Never | 2 (2.2%) |
|  | Missing data | 10 (11.0%) |
| *Did you feel able to say no to any procedures or clinical checks if you wished?* | Always | 47 (51.6%) |
|  | Most of the time | 17 (18.7%) |
|  | Sometimes | 8 (8.8%) |
|  | Not often | 5 (5.5%) |
|  | Never | 4 (4.4%) |
|  | Missing data | 10 (11.0%) |
| *Did the maternity care staff ask for your consent before they carried out vaginal/internal examinations?* | Always | 69 (75.8%) |
|  | Most of the time | 4 (4.4%) |
|  | Sometimes | 5 (5.5%) |
|  | Not often | 1 (1.1%) |
|  | Never | 1 (1.1%) |
|  | Missing data | 11 (12.1%) |
| **Time to care** | | |
| *Did you feel that the maternity care staff spent enough time with you before your baby was born?* | Always | 32 (35.2%) |
|  | Most of the time | 24 (26.4%) |
|  | Sometimes | 12 (13.2%) |
|  | Not often | 11 (12.1%) |
|  | Never | 2 (2.2%) |
|  | Missing data | 10 (11.0%) |
| *Did you feel that the maternity care staff spent enough time with you during the birth?* | Always | 48 (52.7%) |
|  | Most of the time | 16 (17.6%) |
|  | Sometimes | 5 (5.5%) |
|  | Not often | 7 (7.7%) |
|  | Never | 5 (5.5%) |
|  | Missing data | 10 (11.0%) |
| *Did you feel that the maternity care staff spent enough time with you after your baby was born?* | Always | 17 (18.7%) |
|  | Most of the time | 22 (24.2%) |
|  | Sometimes | 15 (16.5%) |
|  | Not often | 18 (19.8%) |
|  | Never | 8 (8.8%) |
|  | Missing data | 11 (12.1%) |
| **Respect, dignity and compassion** | | |
| *Overall, did you feel that the maternity staff treated you with kindness and compassion?* | Always | 39 (42.9%) |
|  | Most of the time | 25 (27.5%) |
|  | Sometimes | 12 (13.2%) |
|  | Not often | 3 (3.3%) |
|  | Never | 1 (1.1%) |
|  | Missing data | 11 (12.1%) |
| *Did you feel the maternity care staff respected your wishes and needs before your baby was born?* | Always | 51 (56.0%) |
|  | Most of the time | 19 (20.9%) |
|  | Sometimes | 7 (7.7%) |
|  | Not often | 3 (3.3%) |
|  | Never | 1 (1.1%) |
|  | Missing data | 10 (11.0%) |
|  | Always | 54 (59.3%) |
| *Did you feel the maternity care staff respected your wishes and needs during childbirth?* | Most of the time | 10 (11.0%) |
|  | Sometimes | 6 (6.6%) |
|  | Not often | 6 (6.6%) |
|  | Never | 4 (4.4%) |
|  | Missing | 11 (12.1%) |
|  | Always | 40 (44.0%) |
| *Did you feel the maternity care staff respected your wishes and needs after your baby was born?* | Most of the time | 17 (18.7%) |
|  | Sometimes | 7 (7.7%) |
|  | Not often | 12 (13.2%) |
|  | Never | 4 (4.4%) |
|  | Missing data | 11 (12.1%) |
|  |  |  |
| **Judgemental interactions** | | |
| *Did any of the maternity care staff speak or use facial expressions or body language in ways that made you feel uncomfortable?* | Always | 1 (1.1%) |
|  | Most of the time | 4 (4.4%) |
|  | Sometimes | 13 (14.3%) |
|  | Not often | 16 (17.6%) |
|  | Never | 46 (50.5%) |
|  | Missing data | 11 (12.1%) |
| *Did any of the maternity care staff make any judgemental or negative comments towards or about you?* | Always | 1 (1.1%) |
|  | Most of the time | 3 (3.3%) |
|  | Sometimes | 11 (12.1%) |
|  | Not often | 11 (12.1%) |
|  | Never | 54 (59.3%) |
|  | Missing | 11 (12.1%) |
| **Mental health needs and support** | | |
| *Did you feel that your mental health was negatively affected following the birth (e.g. low mood, sadness, negative birth memories)?* | Yes | 42 (46.2%) |
|  | No | 38 (41.8%) |
|  | Missing data | 11 (12.1%) |
| *Did you feel able to talk about these feelings? (N=42)* | Yes | 25 (59.5%) |
|  | No | 17 (40.5%) |
| - *Who were you able to talk to about these feelings? Select all that apply. (N=25)* | Midwives | 7 (28.0%) |
|  | Doctors | 2 (8.0%) |
|  | Family | 22 (88.0%) |
|  | Friends | 16 (64.0%) |
|  | Support organisations | 4 (16%) |
| *Did any of the maternity care staff ask you how you were feeling after the birth?* | Yes | 54 (59.3%) |
|  | No | 25 (27.5%) |
|  | Missing data | 12 (13.2%) |
| - *Did they offer to provide you with any help or support for your mental health? (N=54)* | Yes | 37 (68.5%) |
|  | No | 16 (29.6%) |
|  | Missing data | 1 (1.9%) |
| - *Did you access the support that was offered? (N=37)* | Yes | 10 (27.0%) |
|  | No | 27 (73.0%) |
| - *Did the help meet your needs? (N=10)* | Yes, fully | 7 (70%) |
|  | Yes, to some extent | 3 (30%) |
| **Cultural and religious needs and resources** | | |
| *Were you provided with any information or resources which you found culturally appropriate and helpful?* | Yes | 20 (22.0%) |
|  | No | 57 (62.6%) |
|  | Missing data | 14 (15.4%) |
| *Were your religious and/or cultural needs met during your maternity care?* | Yes, fully | 46 (50.5%) |
|  | Yes, to some extent | 18 (19.8%) |
|  | No, not at all | 9 (9.9%) |
|  | Missing data | 18 (19.8%) |
| *Did the maternity care staff have knowledge and understanding of your religious and/or cultural needs?* | Yes, fully | 28 (30.8%) |
|  | Yes, to some extent | 28 (30.8%) |
|  | No, not at all | 15 (16.5%) |
|  | Missing data | 20 (22.0%) |
| *Is having a female care provider important to you?* | Yes | 63 (69.2%) |
|  | No | 11 (12.1%) |
|  | Missing data | 17 (18.7%) |
| - *Were you given the option of a female care provider? (n=63)* | Yes | 26 (41.3%) |
|  | No | 37 (58.7%) |
| *Were you an inpatient in hospital when you were pregnant and/or having your baby?* | Yes | 45 (49.5%) |
|  | No | 30 (33.0%) |
|  | Missing data | 16 (17.6%) |
| - *Were your dietary needs met while you were an inpatient? (n=45)* | Yes, fully | 22 (48.9%) |
|  | Yes, to some extent | 17 (37.8%) |
|  | No, not at all | 6 (13.3%) |
| *During your maternity care, did you have any experiences where your privacy was not respected?* | Always | 1 (1.1%) |
|  | Most of the time | 4 (4.4%) |
|  | Sometimes | 7 (7.7%) |
|  | Not often | 17 (18.7%) |
|  | Never | 51 (56.0%) |
|  | Missing data | 11 (12.1%) |
| *Did you feel that the maternity care staff made any incorrect assumptions about you? (For example, that you could not understand English, or had particular needs).* | Always | 1 (1.1%) |
|  | Most of the time | 2 (2.2%) |
|  | Sometimes | 15 (16.5%) |
|  | Not often | 11 (12.1%) |
|  | Never | 51 (56.0%) |
|  | Missing | 11 (12.1%) |
| *Did you feel that the maternity care staff were less positive or caring towards you, compared to other women?* | Always | 3 (3.3%) |
|  | Most of the time | 8 (8.8%) |
|  | Sometimes | 13 (14.3%) |
|  | Not often | 7 (7.7%) |
|  | Never | 49 (53.8%) |
|  | Missing data | 11 (12.1%) |
